# Ontology representation and analysis of vaccine formulation and administration and their effects on vaccine immune responses

**DOI:** 10.1186/2041-1480-3-17

**Published:** 2012-12-20

**Authors:** Yu Lin, Yongqun He

**Affiliations:** 1 Unit of Laboratory Animal Medicine, Department of Microbiology and Immunology, Center for Computational Medicine and Bioinformatics, University of Michigan Medical School, Ann Arbor, MI 48109, USA

## Abstract

**Background:**

A vaccine is a processed material that if administered, is able to stimulate an adaptive immune response to prevent or ameliorate a disease. A vaccination process may protect the host against subsequent exposure to an infectious agent and result in reduced disease or total prevention of the disease. Vaccine formulation and administration methods may affect vaccine safety and efficacy significantly.

**Results:**

In this report, the detailed classification and definitions of vaccine components and vaccine administration processes are represented using OWL within the framework of the Vaccine Ontology (VO). Different use cases demonstrate how different vaccine formulations and routes of vaccine administration affect the protection efficacy, general immune responses, and adverse events following vaccination. For example, vaccinations of mice with *Brucella abortus* vaccine strain RB51 using intraperitoneal or intranasal administration resulted in different protection levels. As shown in the vaccine adverse event data provided by US FDA, live attenuated and nonliving vaccines are usually administered in different routes and have different local and systematic adverse effect manifestations.

**Conclusions:**

Vaccine formulation and administration route can independently or collaboratively affect host response outcomes (positive protective immunity or adverse events) after vaccination. Ontological representation of different vaccine and vaccination factors in these two areas allows better understanding and analysis of the causal effects between different factors and immune responses.

## Background

A vaccine is any processed material with the function that when administered, it can prevent or ameliorate a disease or disorder in a target organism by inducing or modifying adaptive immune responses specific to the antigens in the vaccine. After the stimulation of a lasting immune response to a protective antigen(s), the host is able to resist the infection of an infectious agent. Vaccine antigens include, for example, suspensions of killed or attenuated microorganisms, or products or derivatives of microorganisms. The most common method of administering vaccines is by injection, but some are given by mouth or nasal spray.

**Figure 1:**
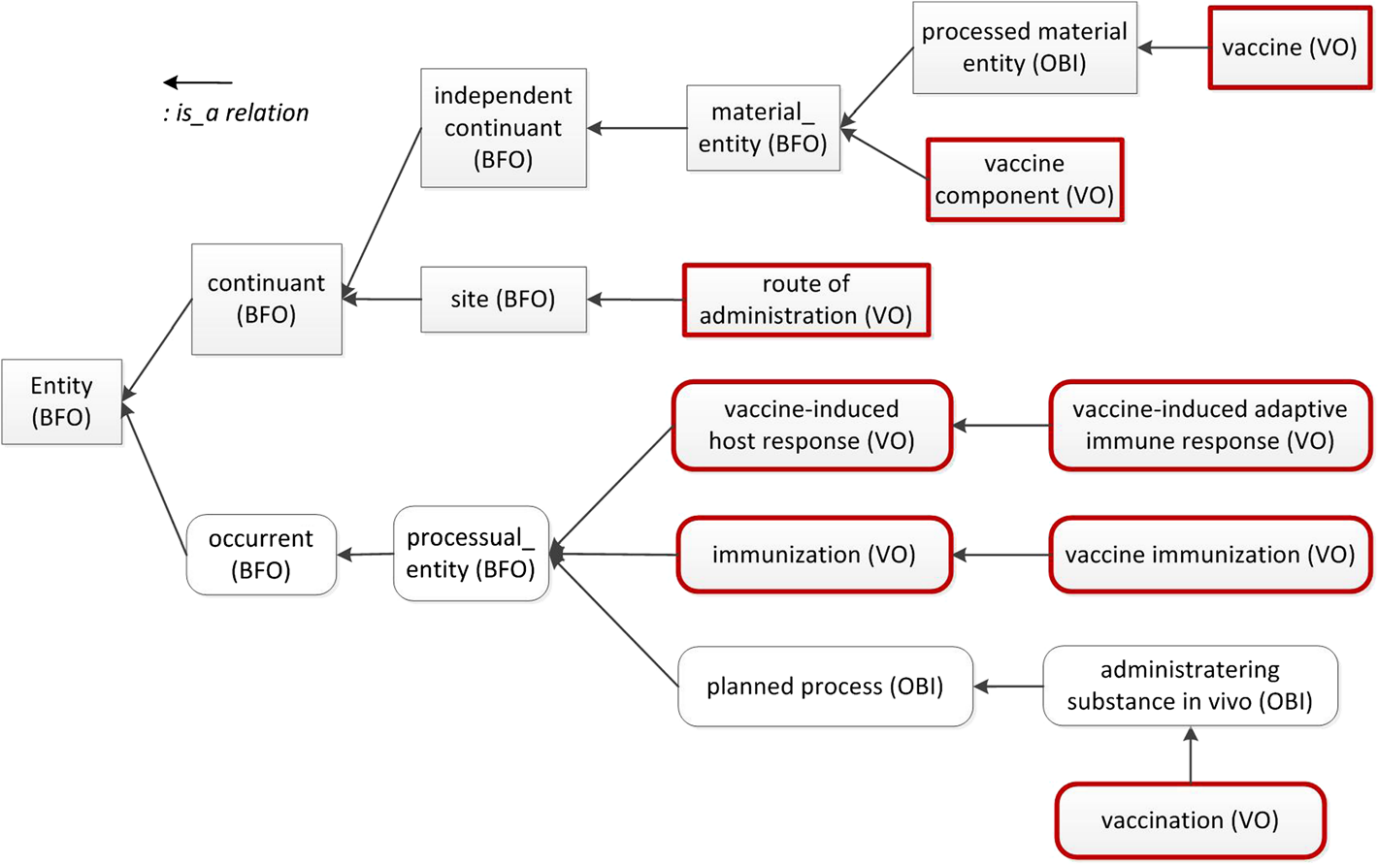
**Selected core VO terms relevant to the presented research.** This figure shows a list of VO core terms relevant to the targeted study. VO is developed under the BFO framework. The VO term ‘vaccine component’ *is a* BFO ‘material entity’. A ‘vaccine’ is an OBI ‘processed material entity’. A ‘route of administration’ *is a* subclass of ‘BFO: site’. ‘vaccine-induced host response’, ‘vaccine-induced adaptive immune response’, ‘immunization’, ‘vaccine immunization’ and ‘vaccination’ are all processes, i.e., ‘processual entity’ in BFO. Note that ‘vaccination’ is asserted as ‘administering substance in vivo’ from OBI. Vaccination is the process of administering a vaccine into a host. It differs from vaccine immunization that is a biological process that starts at vaccination and results in an outcome of the host getting immunized.

The domain of the vaccine and vaccination research spans multiple areas: the pathogen, vaccine preparation, vaccine administration, vaccine-induced immune response, vaccine safety and efficacy. The Vaccine Ontology (VO) is a community-based ontology in the field of vaccine and vaccination [1]. VO has been developed under the framework of Basic Formal Ontology (BFO 1.1) [2], and it uses the Relation Ontology (RO) [3] to represent commonly used relations. VO formally represents various vaccines including those that are licensed, in clinical trial, or just proven effective in laboratory research. In addition, VO ontologically represents different vaccine components and how different components exist in any specific vaccine. VO also captures the knowledge of vaccination, immunization and the vaccine-host interactions. Figure 1 represents selected top level core VO terms relevant to this study; it includes vaccine, vaccine component, route of administration, vaccination, immunization, and vaccine-induced host response. Because of their importance, vaccine-induced immune responses and vaccine protection against targeted diseases or pathogens are emphasized in VO. In addition to vaccine-specific terms, VO has also included terms imported from more than 10 existing ontologies, such as the Chemical Entities of Biological Interest (CHEBI) [4], the Ontology for Biomedical Investigations (OBI) [5], and the Infectious Disease Ontology (IDO) [6]. These reliable biomedical ontologies provide higher level terms or important entities that are used in VO, and reusing them supports ontology interoperability.

Vaccine-induced host responses can be protective immunity (desired response) or adverse events (undesired responses). The induction of protective immunity is the outcome of vaccine efficacy. The appearance of adverse events is often due to the side effects of a vaccine and is an important topic of vaccine safety. To study the efficacy and safety of a vaccine, the knowledge of vaccine formulation and administration is very important. We hypothesize that the differences in vaccine formulation and administration will change the induction of vaccine-induced protective immunity and adverse events. This hypothesis has not been systematically addressed at the ontological level. In this report, we first represent various vaccine formulations and routes of vaccine administration in VO, and then discuss how different vaccine formulations and vaccination routes influence the outcomes of vaccination, including protective immune responses and adverse events.

## Results

In what follows, italics are used to refer to ontology relation terms, logical or textual definitions of ontology terms. Single quotation marks ‘ ’ are used to cite other class and instance level ontology terms.

### Representing vaccine components in VO

**Figure 2:**
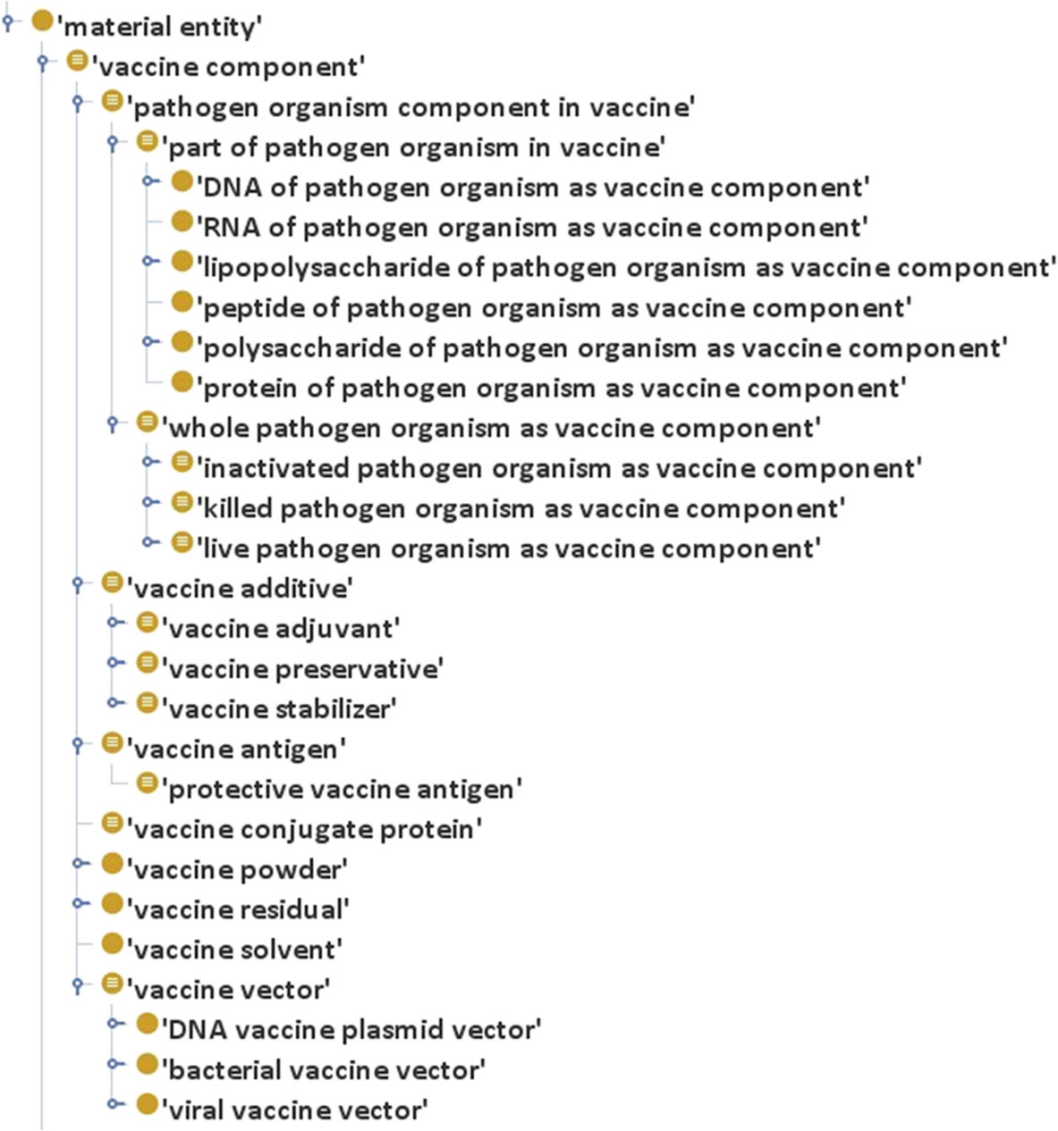
Vaccine component represented in VO.

The final form of a vaccine is often a mixture of different ingredients. In the manufacturing of licensed vaccine products, the vaccine formulation refers to a uniform mixture of all vaccine components into a single vessel. A vaccine is a mixture of different components including vaccine antigen, adjuvant, and buffer. In VO, a ‘vaccine component’ is defined as a material entity that is part of a vaccine. The logical definition of ‘vaccine component’ in VO is: *‘material entity’ and (part_of some ‘vaccine’).* Different ingredients of vaccines were classified into subclasses under the class ‘vaccine component’ (Figure 2). Selected vaccine components related to this study are described below:

**‘Pathogen organism vaccine component’:** a vaccine component that is either a pathogen organism or derived from a pathogen organism. This term is logically defined in VO as: *'vaccine component' and (organism or ('is part of' some organism))*.

The pathogen organism used for vaccine development is an output of the vaccine preparation process, which includes propagation of the pathogen in a culture medium, followed by isolation and purification of the organism. Live attenuated or killed virulent pathogens can be used as a key component of a vaccine. A killed or inactivated vaccine is produced by killing the pathogenic microbe with chemicals, heat or radiation. A live attenuated vaccine contains living microbe that has been weakened (attenuated) in a laboratory setting so it can no longer cause severe disease. A vaccine that has live attenuated pathogen as a component is defined as a ‘live attenuated vaccine’ in VO. Similarly, a vaccine that has killed or inactivated pathogen as a component is defined as a ‘killed or inactivated vaccine’. Vaccines use proteins, peptides, lipopolysaccharides, or polysaccharides of a pathogen as their components defined as ‘subunit vaccine’ in VO. Vaccines use the DNA or RNA sequence of a pathogen organism as component is a DNA or RNA vaccine.

**‘Vaccine antigen’:** An immunogenic antigen in a vaccine. A vaccine antigen is typically a modified or partial form of a virus, bacterium, or toxin that causes a disease. VO’s logical definition is: *‘vaccine component’ and has role some ‘antigen role’*. The vaccine antigen is able to induce an immune response. An example of a vaccine antigen is a protein from a pathogen organism as described above. Note that some subclasses of ‘pathogen organism vaccine component’ bears ‘vaccine antigen role’ such as some proteins, peptides, lipopolysaccharides and polysaccharides used for vaccine component. However, some other ‘pathogen organism vaccine component’ may not bear the ‘vaccine antigen role’. For example, the host may not produce any immune response to some component (*e.g.*, water, some metabolites, and some peptides) of a pathogen organism.

**‘Vaccine conjugate protein’:** is a protein that is chemically conjugated to a vaccine antigen and is used to enhance vaccine-induced immune response. A vaccine that uses a conjugate protein called a conjugate vaccine. The conjugate protein may help to induce the T-cell-dependent immune response and confer immune memory to a vaccine antigen. The logical definition for ‘vaccine conjugate protein’ in VO is: *'vaccine component' and (bearer_of some 'vaccine conjugate role')*.

**‘Vaccine additive’:** is a material added to the antigenic component by the vaccine manufacturer for a specific purpose, such as stabilizing the final product as in vaccine stabilizer, strengthening vaccine induced immune response as in vaccine adjuvant, and preventing serious adverse effect such as *Staphylococcus* infection as in vaccine presevatives. The term is logically defined in VO as: *'vaccine component' and (bearer_of some 'vaccine additive role')*. Vaccine additives include adjuvants, preservatives and stabilizers, as well as materials that are added to affect pH and isotonicity [7].

An important vaccine additive is ‘**vaccine adjuvant**’: a vaccine additive incorporated into a vaccine to enhance the immunogenicity of vaccine antigens. Adjuvants can function as immunostimulants, vehicles, and carriers. In general, the adjuvant effect can be divided into two principal components: (1) antigen depot and delivery and (2) immune potentiation by targeting antigens to antigen presenting cells (APC) [8,9]. Vaccine delivery systems are generally particulates, *e.g.*, emulsions, microparticles, immune stimulating complexes (ISCOMS) and liposomes. The particulate characteristics facilitate uptake by APCs and transport to secondary lymph organs, resulting in the induction of immune responses [9]. The logical definition of this term in VO is: *'vaccine additive' and ('has role' some 'vaccine adjuvant role')*.

**‘Vaccine vector’**: a vector that is used in a genetically engineered vaccine to transport and express genes coding for antigens into the body to induce an immune response. Examples of a vaccine vector include a plasmid vector for the generation of a DNA vaccine and a weakened or killed version of a virus or bacterium that cannot cause a disease in hosts (*e.g.*, human). VO logically define vaccine vector as *'vaccine component' and (bearer_of some 'vaccine vector role')*.

Different vaccine components may share similar functions. For example, vaccine adjuvant and vaccine conjugate protein both help to induce stronger immune responses. A vaccine conjugate protein is chemically bound to a vaccine antigen, whereas, a vaccine adjuvant is just mixed with the vaccine without any chemical conjugation. A vaccine conjugate protein may by itself induce host immune response, but a vaccine adjuvant usually does not. Another example is vaccine vector and vaccine adjuvant, both of which convey a carrier’s function. A vaccine vector carries the genetic material that encodes for a protein antigen. However, a vaccine adjuvant delivery system carries the vaccine antigen per se. These important differentiations are captured using definition of roles. For example, the definition of ‘vaccine vector role’ in VO is “*a biological vector role that is realized by the process of transmitting genetic material encoding vaccine antigen to a vaccinee, when introduced into the vaccinee, it results in the in situ production of antigen in the vaccinee*” [10].

Several roles including ‘vaccine additive role’, ‘vaccine adjuvant role’, ‘vaccine stabilizer role’, ‘vaccine preservative role’, ‘vaccine vector role’ and ‘vaccine conjugate role’ are generated in VO. Bearers of these roles are corresponding vaccine components. Through the definition of roles, the usages and purposes of different vaccine components become clearly defined in VO.

### Vaccine administration

The route of a vaccine administrated into a vaccinee is another key factor for evaluating a vaccine’s efficacy and safety. For example, if administered through nose, a vaccine may induce stronger mucosal immune response but weaker systematic immunity than that through injection. Based on He’s previous work of term ‘vaccination’ as a child term of ‘administering substance in vivo’ [11], more detailed classification of subtypes of vaccination were given in this paper.

There are two ways to differentiate different vaccination: 1) based on frequency; and 2) based on entry location. For the first categorization, there are ‘primary vaccination’, and ‘boost vaccination’. ‘Boost vaccination’ has ‘secondary vaccination’, ‘tertiary vaccination’, and ‘quaternary vaccination’ as its children. The ontological relation between those vaccinations ‘*is preceded by’* describes a temporal link between classes of processes [3]. For example, in VO, the ‘boost vaccination’ *is preceded by* ‘primary vaccination’, which indicates that if a vaccinee has his/her boost vaccination, he/she must have had a primary vaccination some time earlier.

**Figure 3:**
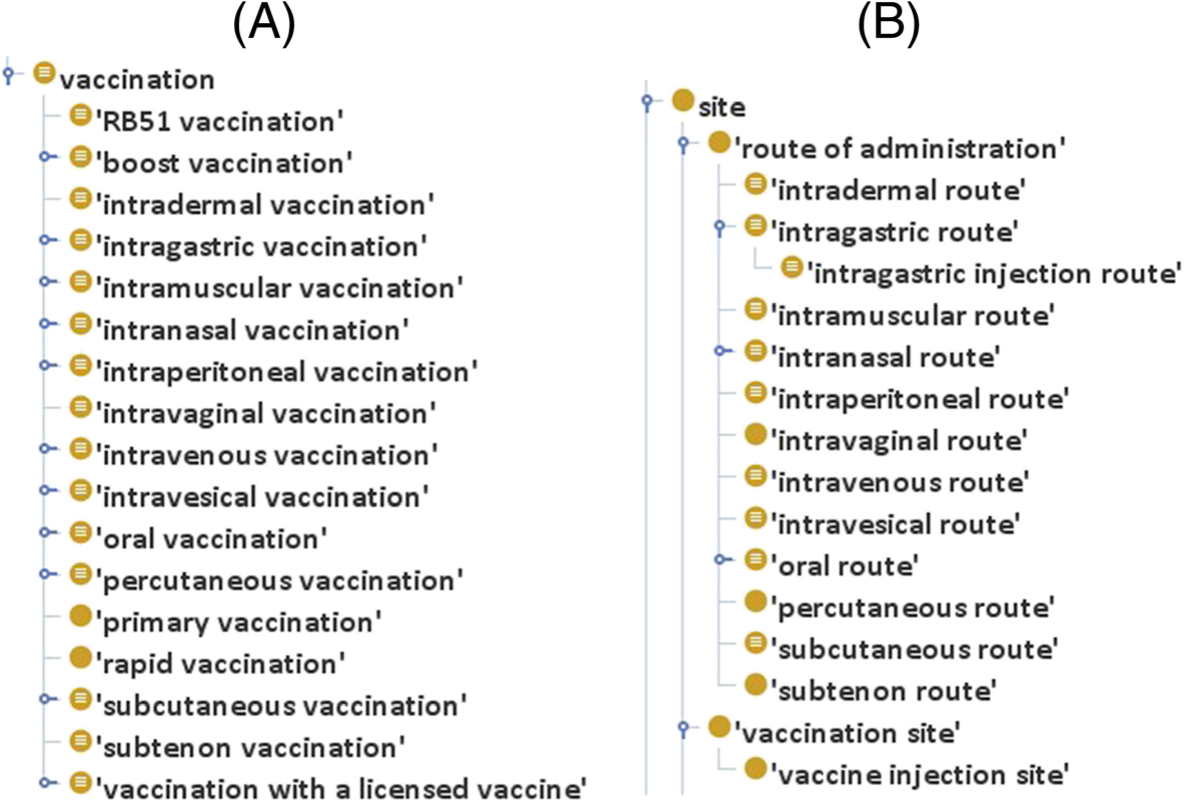
VO classification of (A) vaccination (B) route of administration.

Another classification of vaccinations is based on the entry locations of a vaccine administration. Specifically, a vaccination can be classified as intramuscular (i.m.), intraperitoneal (i.p.), intragastric (i.g.), intradermal (i.d.), subcutaneous (s.c.), percutaneous, intravenous (i.v.), intravesical, oral, intranasal (i.n.) vaccination and many others (Figure 3A). The ontological definition for this subtype of vaccination utilizes the relation ‘*unfolds_in*’ and a ‘route of administration’. For example, an ‘intravenous vaccination’ is defined as *‘vaccination and unfolds in some intravenous route’*. NCI thesaurus (http://ncit.nci.nih.gov/) hosts the term ‘route of administration’ from Clinical Data Interchange Standards Consortium (CDISC) terminology (http://www.cdisc.org). We adopted and refactored this term in VO. The term ‘route of administration’ is asserted as a subclass of BFO:site (Figure 3B). It means the fiat path where the vaccine is being taken into body, which starts with the vaccine entry point of body and ends at the site of action in the body. It is asserted with a description logical constrain: *‘is located in’ some ‘gross anatomical part’*. Using this parent term, a subclass term oral route is logically defined as: *‘route of administration and is located in some (mouth or stomach)’*.

During a vaccine administration process, the site of vaccination is often needed to be recorded. Term ‘vaccination site’ and ‘vaccine injection site’ have been created for this purpose. Both the terms refer to a site of the host body where a vaccine enters into host. A ‘vaccine injection site’ explicitly refers to where injection needle or needles pierced through, and a vaccination site may refer to a location of the body (*e.g.*, nose or skin) where a needle free delivery system delivers vaccine. The ‘vaccination site’ is modeled as part of an administration route. Specifically, an administration route is the fiat path of an administration, and the vaccination site is the entrance point of an administration route. By this treatment, vaccination through oral route or intranasal route has no vaccine injection site but has a vaccination site.

### Effects of formulation and administration to vaccine-induced immune responses

The immune responses induced by a vaccine may be affected by many factors, including the type of vaccine, vaccine dose, route of administration and the presence of an adjuvant. Host factors such as age, nutritional factors, genetics, and coexisting disease may also affect the response [12]. Here we show how different vaccine formulations and routes of administration will affect the immune responses in the host after vaccination.

### Effects of formulation to vaccine-induced immune responses

Many examples can be used to demonstrate the important role of formulation to the vaccine-induced immune responses. For instance, killed or live attenuated vaccines turn to induce different immune responses. Live attenuated vaccines usually induce superior protection against subsequent challenge [13]. However, they often present safety risks. In contrast, vaccines based on killed whole pathogens, although safe, typically induce weak cellular immunity [14]. One primary reason is that killed pathogen cannot infect the host and replicate, a process that typically stimulates strong host response. Another reason is that the physical or chemical treatments used for killing pathogens may result in the denaturation of the antigen proteins or damage of the organism [15].

*Brucella abortus* strain RB51 is a live attenuated vaccine against cattle brucellosis [16]. It has been licensed for commercial use in the US and many other countries. Live RB51 can induce both cell-mediated and humoral immunity against virulent *Brucella* infection. However, killed whole pathogen organism of RB51 fails to induce protective cell-mediated immunity [17]. Another example is influenza vaccines. Currently there are both trivalent live attenuated or inactivated influenza vaccines available in the market. Although both live and inactivated influenza vaccines induce similar humoral responses, only live attenuated vaccines induce diverse T-cell responses in young children [18].

Killed vaccines typically induce Th2 immune response. One approach for improving the performance of killed vaccine is to use an effective vaccine adjuvant. To avoid strong adverse effects and still induce strong immune response, it is often feasible to use gamma-irradiated pathogen that cannot replicate but remain metabolically active for a period of time to induce antigen-specific immune response as live attenuated vaccine [14,19].

The usage of conjugate vaccines is another example of the effect of formulation to vaccine-induced immune response. Polysaccharide (PS) antigens of many pathogenic bacteria induce only weak and short immune response, especially in young children populations, due to their immunological naivety and a degree of immunoincompetence condition [20]. The response to a capsular PS is T-cell-independent, meaning that B lymphocytes proliferate and produce antibody without the help of T cells [21]. Conjugate vaccines are formed by linking capsular polysaccharides to a protein carrier, such as tetanus toxoid, diphtheria toxoid, or *Corynebacterium diphtheriae* cross-reactive material (CRM197). Conjugate vaccines may induce T-cell-dependent immunologic responses that confer immune memory. In the USA, three meningococcal vaccines are approved by FDA: conjugate vaccine Menactra® by Sanofi Pasteur, conjugate vaccine Menveo® by Novartis Vaccines and Diagnostics, and polysaccharide vaccine Menomune -A/C/Y/W-135® (MPSV4) by Sanofi Pasteur. All these vaccines are using meningococcal polysaccharide from serogroups A,C,W-135 and Y as vaccine antigens. Menactra® and Menveo®, but not polysaccharide vaccine Menomune® are able to induce high immunogenicitiy and immunologic memory especially in young children population [7].

### Effect of vaccine administration route to protective immunity

The route of administration also plays a role in the efficacy of a vaccine. A vaccine protection investigation includes three processes: vaccination, pathogen challenge, and vaccine protection efficacy assessment. A measurement of vaccine efficacy is often assessed by host survival for the pathogens (*e.g.*, Influenza virus) which kill the infected host (*e.g.*, mouse) [22]. Many pathogens such as virulent *Brucella*[23] cannot kill infected host. In this case, different methods have to be generated to measure the vaccine efficacy. For example, the measurement of *Brucella* colony forming units (CFU) in spleens and/or livers of infected or noninfected mice measurement is established to measure diminished replication in a vaccinated host than that in unvaccinated host [23]. After a laboratory animal model is set up to measure vaccine efficacy, it is possible to test if a change of the administration route would change the vaccine efficacy.

**Figure 4:**
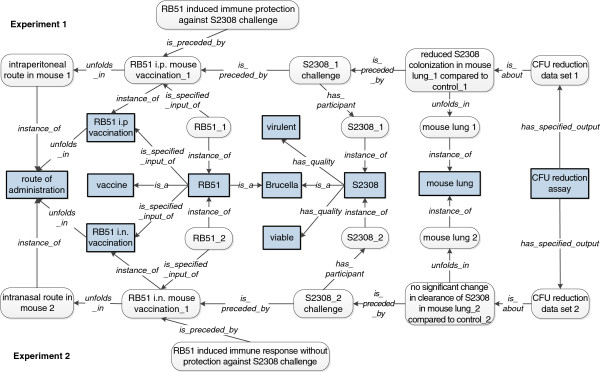
**Modelling the RB51-induced different immune response with different route administration.** The upper part of this figure models the experimental results that interperitoneal RB51 vaccination in mice induced protection against virulent strain S2308 challenge in the lung [25]. The lower part of this figure models another study which showed that intranasal RB51 vaccination was unable to protect against virulent strain 2308 challenge in the lung as investigated in experiment 2 [26]. Filled square boxes indicate class level terms. Boxes with round corners represent individual level data collected from papers.

As a case study, we ontologically modeled two reported studies of vaccination-challenge experiments with different vaccination routes but the same *Brucella* vaccine in an established mouse model (Figure 4). RB51 is a licensed live attenuated *Brucella abortus* vaccine, and the BALB/c mouse model is a well-established laboratory animal model for *Brucella* vaccine research [16,24]. As shown in one study, when RB51 was used to vaccinate a group of BALB/c mice through the intraperitoneal route (i.p.), the vaccination induced protection in the mouse lung against i.p. challenge of the virulent *B. abortus* strain S2308 [25]. However, in another study, when the intranasal route (i.n.) was used for RB51 vaccination using the same BALB/c mouse model, the vaccinated mice were not able to protect against i.n. challenge of virulent *Brucella abortus* strain 2308 in the lung [26]. The data extracted from each experiment in papers are modelled ontologically using individuals in VO (Figure 4). Specifically, RB51 induced immune response with/without protection against S2308 in lung *is preceded by* the RB51 vaccination process, which *unfolds in* the ‘route of administration’ (*e.g.,* intraperitoneal route or intranasal route). RB51 is the specific input of the RB51 vaccination and S2308 is a participant of ‘S2308 challenge’. ‘CFU reduction assay’ has specific output ‘CFU reduction data set’, which *is about* the colonization or clearance of S2308 in mouse. In experiment 1 [25], the mouse was vaccinated through i.p. route, which led to ‘RB51-induced immune protection against S2308 challenge’. Proceded by the i.p. vaccination, the ‘S2308_1 challenge’ leads to the ‘reduced S2308 colonization in mouse lung_1 when compared to control_1’ process, revealed by the ‘CFU reduction data set_1’ (*is_about*). Similarly, in another experiment 2 [26], the ‘RB51-induced immune response without protection against S2308 challenge’ was proceded by a ‘RB51 i.n. mouse vaccination_1’. The vaccination is followed by ‘S2308_1 challenge’ and then ‘no significant change in clearance of S2308 in mouse lung when compared to control_2’, revealed by the ‘CFU reduction data set_2’.

In summary, the RB51 vaccination-challenge studies clearly demonstrate that different vaccination routes may change the outcome of the vaccine efficacy even though the same vaccine is used. Therefore, it is critical to optimize vaccination conditions to achieve the best vaccination results.

### Combinatorial effects of vaccine formulation and administration routes to vaccine adverse event profiles

Using the information from the package inserts of 53 licensed US vaccines listed by the US FDA, an analysis of the effects of vaccine formulation and vaccination routes on the advance event occurrences was conducted. In total, 53 licensed vaccines are used in the US market. These 53 vaccines are classified into two groups: ‘live attenuated vaccine’ and ‘nonliving vaccine’. Applying the VO’s definition of ‘live vaccine’ and ‘live attenuated vaccine’, all the vaccines that do not contain live microbe as vaccine component are considered nonliving vaccines, which includes inactivated vaccines, subunit vaccines, and conjugate vaccines. Local reaction is defined as any adverse event that occurs at the vaccination site after the vaccination. Systematic reactions are the adverse events unfolding in the whole body or any anatomical system in human body.

Out of 53 vaccines, 13 (24.5%) are live attenuated vaccines (Table 1). The routes of administration of these live attenuated vaccines are: subcutaneous (7, 53.8%), oral (3, 23%), intranasal (1, 7.7%), percutaneous (2, 15.4%) and intravesical (1, 7.7%). Among 40 nonliving vaccines (Table 2), 38 (95%) are administrated intramuscularly, and 3 (7.5%) are administrated subcutaneously. The inactivated vaccine IPOL (Poliovirus Vaccine Inactivated manufactured by Sanofi Pasteur) is administrated either intramuscularly or subcutaneously. No local reactions were reported after oral administration. The most common local adverse events following vaccine injection are pain, induration and tenderness at the injection site. Intranasal administration may result in nose symptoms. Systematic adverse events were reported in the package inserts of all vaccines (Table 1 and Table 2).

**Table 1:** Comparison of adverse event following vaccination of 13 live attenuated human vaccines licensed in USA

**Vaccine trade name**	**VO_ID**	**Route of administration**	**Local reactions (Y/N)**	**Systematic reactions (Y/N)**
Adenovirus Type 4 and Type 7 Vaccine	VO_0000096	oral	N	Y
Rotarix	VO_0010738	oral	N	Y
RotaTeq	VO_0000097	oral	N	Y
BCG Vaccine	VO_0003240	percutaneous	Y	Y
ACAM2000	VO_0000003	percutaneous	Y	Y
TICE BCG	VO_0000103	intravesical	Y	Y
FluMist® Quadrivalent	VO_0000044	i.n.	Y	Y
Attenuvax	VO_0000007	s.c.	Y	Y
M-M-R II	VO_0000069	s.c.	Y	Y
ProQuad	VO_0000091	s.c.	Y	Y
MERUVAX II	VO_0000073	s.c.	Y	Y
Varivax	VO_0000119	s.c.	Y	Y
YF-Vax	VO_0000121	s.c.	Y	Y
Zostavax	VO_0000124	s.c.	Y	Y

**Table 2:** Comparison of adverse event following vaccination of 40 nonliving human vaccines licensed in USA

**Vaccine trade name**	**VO_ID**	**Route of administration**	**Local reactions (Y/N)**	**Systematic reactions (Y/N)**
Biothrax	VO_0000014	i.m.	Y	Y
Diphtheria & Tetanus				
Toxoids Adsorbed	VO_0000033	i.m.	Y	Y
Tripedia	VO_0000112	i.m.	Y	Y
Infanrix	VO_0000064	i.m.	Y	Y
Daptacel	VO_0000029	i.m.	Y	Y
Pediarix	VO_0000082	i.m.	Y	Y
Kinrix	VO_0000067	i.m.	Y	Y
Pentacel	VO_0000084	i.m.	Y	Y
ActHIB	VO_0000004	i.m.	Y	Y
Hiberix	VO_0010715	i.m.	Y	Y
Liquid PedvaxHIB	VO_0010723	i.m.	Y	Y
Comvax	VO_0000028	i.m.	Y	Y
Havrix	VO_0000052	i.m.	Y	Y
VAQTA	VO_0010745	i.m.	Y	Y
TWINRIX	VO_0000113	i.m.	Y	Y
Engerix-B	VO_0010711	i.m.	Y	Y
RECOMBIVAX HB	VO_0010737	i.m.	Y	Y
Gardasil	VO_0000049	i.m.	Y	Y
CERVARIX	VO_0011559	i.m.	Y	Y
Influenza A (H1N1) 2009 Monovalent Vaccine (CSL Limited)	VO_0000348	i.m.	Y	Y
Influenza A (H1N1) 2009 Monovalent Vaccine (ID Biomedical Corporation of Quebec)	VO_0002499	i.m.	Y	Y
Influenza A (H1N1) 2009 Monovalent Vaccine (Novartis Vaccines and Diagnostics Limited)	VO_0000081	i.m.	Y	Y
Influenza A (H1N1) 2009 Monovalent Vaccine ( Sanofi Pasteur Inc.)	VO_0000160	i.m.	Y	Y
Influenza Virus Vaccine, H5N1	VO_0000065	i.m.	Y	Y
AFLURIA	VO_0000006	i.m.	Y	Y
Agriflu	VO_0001126	i.m.	Y	Y
FluLaval	VO_0000043	i.m.	Y	Y
Fluarix	VO_0000045	i.m.	Y	Y
Fluvirin	VO_0000046	i.m.	Y	Y
Fluzone	VO_0000047	i.m.	Y	Y
Ixiaro	VO_0011558	i.m.	Y	Y
JE-Vax	VO_0000066	s.c.	Y	Y
MENVEO	VO_0001246	i.m.	Y	Y
Menactra	VO_0000071	i.m.	Y	Y
Menomune	VO_0000072	s.c.	Y	Y
Pneumovax 23	VO_0000088	i.m.	Y	Y
Prevnar	VO_0000090	i.m.	Y	Y
Prevnar 13	VO_0001244	i.m.	Y	Y
IPOL	VO_0000054	i.m. or s.c.	Y	Y
IMOVAX	VO_0010716	i.m.	Y	Y

This result indicates that formulation of vaccine and route of administration interactively affect immune response. The vaccine formulation is likely a determinant factor to decide the route of administration. Since live vaccines have the ability to invade into the host, migrate into different areas of the body, and replicate, they can be administrated using various routes such as subcutaneous, oral, and intranasal. Most of nonliving vaccines are delivered by intramuscular injection which allows the dissemination of the antigens to the antigen presentation cells relatively easily. To promote the antigen presentation, most nonliving vaccines need adjuvant or effective carrier component in their formulation. Since oral administration does not relate with local reaction, the local adverse reactions after vaccination may relate with the use of needle for vaccine injection.

## Discussion

The formulation and routes of administration of vaccines have been represented using OWL in VO. The classification and definitions of their subclasses have been given in this paper. The presented use cases support the hypothesis that there are combinatorial effects of formulation and administration route on the outcomes of vaccination including protective immune responses and vaccine adverse events. The ontological and semantic modeling of vaccine formulation and administration is a starting point for systematically analyzing various vaccine data in different resources with the goal to identify new knowledge. It is noted that some other factors such as vaccinee’s health condition, vaccine dose or vaccination frequency would also have an impact on the outcome of vaccination. Those factors could be later further included to current modelling and provide more comprehensive vaccine effect analysis.

Live attenuated vaccines can usually induce stronger protection, but they are considered unsafe in different scenarios. Killed vaccines or vaccines using part of a pathogen organism are considered safer but usually require additives (*e.g.* adjuvant) to boost host immune response to vaccine antigens. However, in the formulation of vaccines, less additives and residuals is more benefit for vaccinees. For example, using mutant non-toxic CRM_197_ (as in Menveo®) instead of toxic diphtheria toxoid (as in Menactra®) as conjugate protein will reduce the use of formaldehyde or other detoxifiers [27].

How risky is it to use a live attenuated vaccine in terms of vaccine safety? Our case study shows that an orally administrated live attenuated vaccine induces systematic adverse events and no local adverse events. However, the vaccine formulation is not the only determinant for vaccine-induced immune responses. As demonstrated in our study of the 53 licensed vaccines in the US, the live and nonliving vaccines are administrated via different routes. No example can be used to compare vaccine-induced adverse event or immune response if considering differences of only one variable: the vaccine formulation. One exception is for live vs. nonliving Japanese encephalitis vaccines. Both vaccines contain the same vaccine antigen and are administrated in the same injection route. However, they are licensed and used in different countries, thus well-controlled valuable data for systematic comparison cannot easily be obtained. To better identify the differential immune responses by live vs. nonliving licensed Japanese encephalitis vaccines, a systematic analysis of protective immune responses and adverse events on a global scale is needed.

It is relatively straightforward to link local vaccine-induced adverse events (*e.g.*, redness and local pain) with the vaccine and the administration route. However, it is often difficult to assert that systematic adverse events are induced by the administration of a vaccine. Asserting the causal relations between systematic adverse events and vaccine administrations is rather another topic, which is out of the scope of this paper and shall be addressed by the Ontology of Adverse Event (OAE) [28]. Finally, a system bioinformatics approach that integrates ontology modeled with analysis of gene expression data, reported literature results, and vaccine adverse event case reports shall provide the solution for answering the questions.

## Conclusions

This is the first time that we report an OWL modeled of vaccine formulation and administration and how they affect the vaccine-induced immune responses. Various use cases were used to demonstrate the correlative relation between the formulation/administration and the immune response outcome. The application of the ontological modeled will lead to better understanding of the causal effects between immune outcomes and different factors in vaccine preparation and vaccination.

## Methods

### Ontology editing

The VO development follows the OBO Foundry principles, including openness, collaboration, and use of a common shared syntax [29]. The format of VO ontology is W3C standard Web Ontology Language (OWL2) (http://www.w3.org/TR/owl-guide/). For this study, many new terms and logical definition were added into existing VO using the Protégé 4.1 OWL ontology editor (http://protege.stanford.edu/). VO project website is: http://www.violinet.org/vaccineontology.

### Data sources for ontology development

Reference books are the main resource for classification and definition of vaccine formulation and administration [7,30]. Definitions of terms related in this paper were generated and reviewed by experts from the VO development team. A mail listing of VO developers was used to discuss and achieve agreement on logical definitions. The USA CDC pink book serves as a source for populating information related with U.S. licensed human vaccines. The vaccine ingredients used in U.S. licensed vaccines were manually extracted from an appendix table in the CDC pink book. The VIOLIN vaccine database, a web-based central resource for vaccine-related research data across various human pathogens [31], is another source for populating VO. Data used for use case analyses were manually extracted from: (i) PubMed papers and (ii) package inserts of licensed vaccines listed in US Food and Drug Administration (FDA) website [32].

### Use case studies

Use case studies were designed to address the following questions: how the differences of vaccine ingredients and/or administration routes influence vaccine efficacy or adverse event reactions? To simplify the story, the effects of other factors, such as patient health condition, vaccine dose, and vaccination frequency, are not analyzed in this study. VO is used for classification of vaccine types in the use cases.

## Competing interests

The authors declare that they have no competing interests.

## Authors’ contributions

YL: Project design, ontology development, data interpretation, and drafting of manuscript. YH: Project design, ontology development, data interpretation, and drafting of manuscript. Both authors read and approved the final manuscript.
